# Supplementary material for: Image- and Fluorescence-Based Test Shows Oxidant-Dependent Damages in Red Blood Cells and Enables Screening of Potential Protective Molecules
Source: Int J Mol Sci. 2021 Apr 20;22(8):4293. doi: 10.3390/ijms22084293 (PMC8074894; doi:10.3390/ijms22084293)
Supplement: Supplementary file 1 [file ijms-22-04293-s001.zip › ijms-1147132-supplementary.pdf]

International Journal of *Molecular Sciences*

Supplementary Material

Image- and Fluorescence-based Assay to Test Red Blood Cells Sensitivity to Oxidative Stress and Screen Potential Protective Molecules

Manon Bardyn [1], Jérôme Allard [1,2], David Crettaz [1], Benjamin Rappaz [3], Gerardo Turcatti [3], Jean-Daniel Tissot [4] and Michel Prudent [1,4,5*]

1 Laboratoire de Recherche sur les Produits Sanguins, Transfusion Interrégionale CRS, Epalinges, Switzerland
2 Département de Génie Chimique, École Polytechnique de Montréal, Montréal, Québec, Canada
3 Biomolecular Screening Facility (BSF), Ecole Polytechnique Fédérale de Lausanne (EPFL), Lausanne, Switzerland
4 Faculté de Biologie et de Médecine, Université de Lausanne, Lausanne, Switzerland
5 Center for Research and Innovation in Clinical Pharmaceutical Sciences, Institute of Pharmaceutical Sciences of Western Switzerland, University Hospital and University of Lausanne, Lausanne, Switzerland

* Correspondence: Michel.Prudent@itransfusion.ch; Tel.: +41-21-333-16-70 (M.P.)

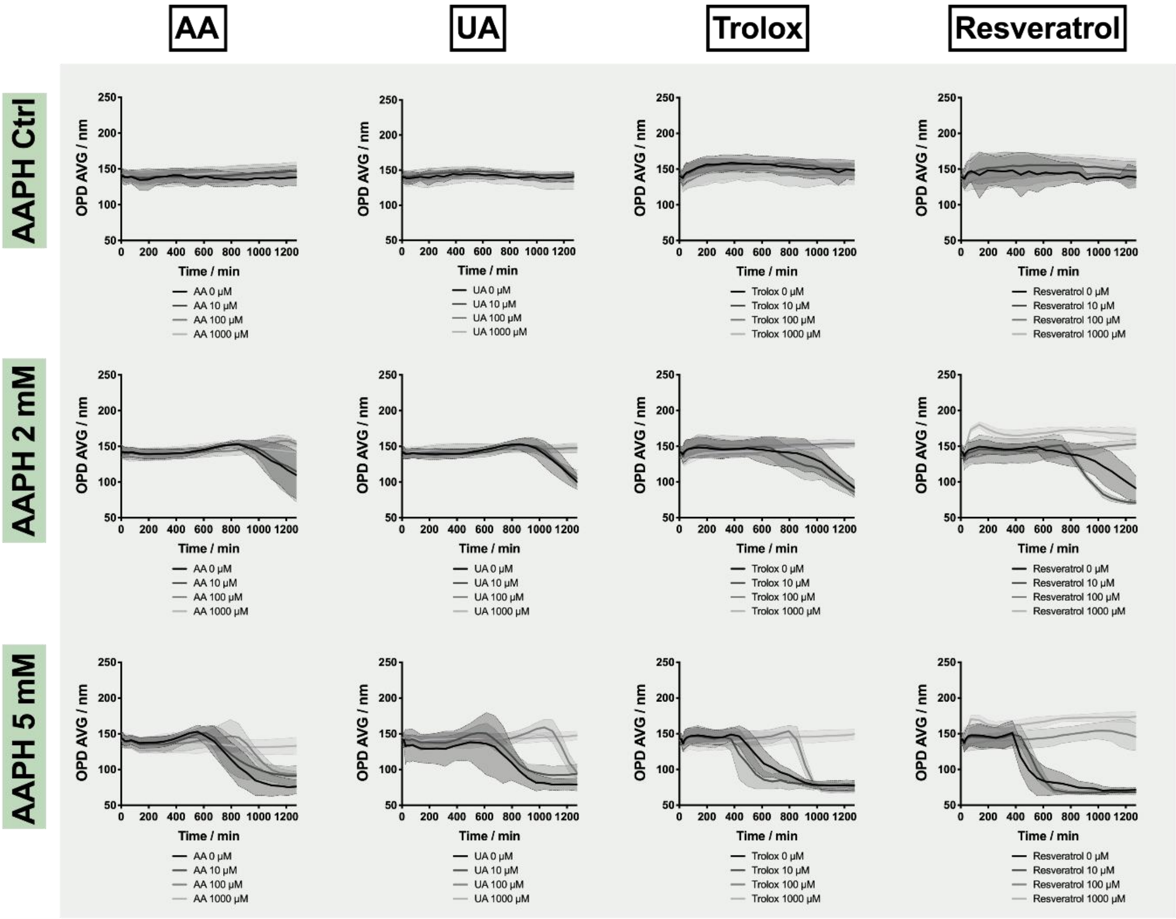

Figure 1. Summary Panel of the Effects on Red Blood Cells (RBCs) of 0, 10, 100 and 1000 µM Ascorbic Acid (AA), Uric Acid (UA), Trolox and Resveratrol against AAPH at Different Concentrations. Timelapse Curves of Average of the Optical Path Difference Distribution (OPD AVG) Parameter Acquired by Digital Holographic Microscopy (DHM). Mean of 3 Red Cell Concentrates with Standard Deviation.

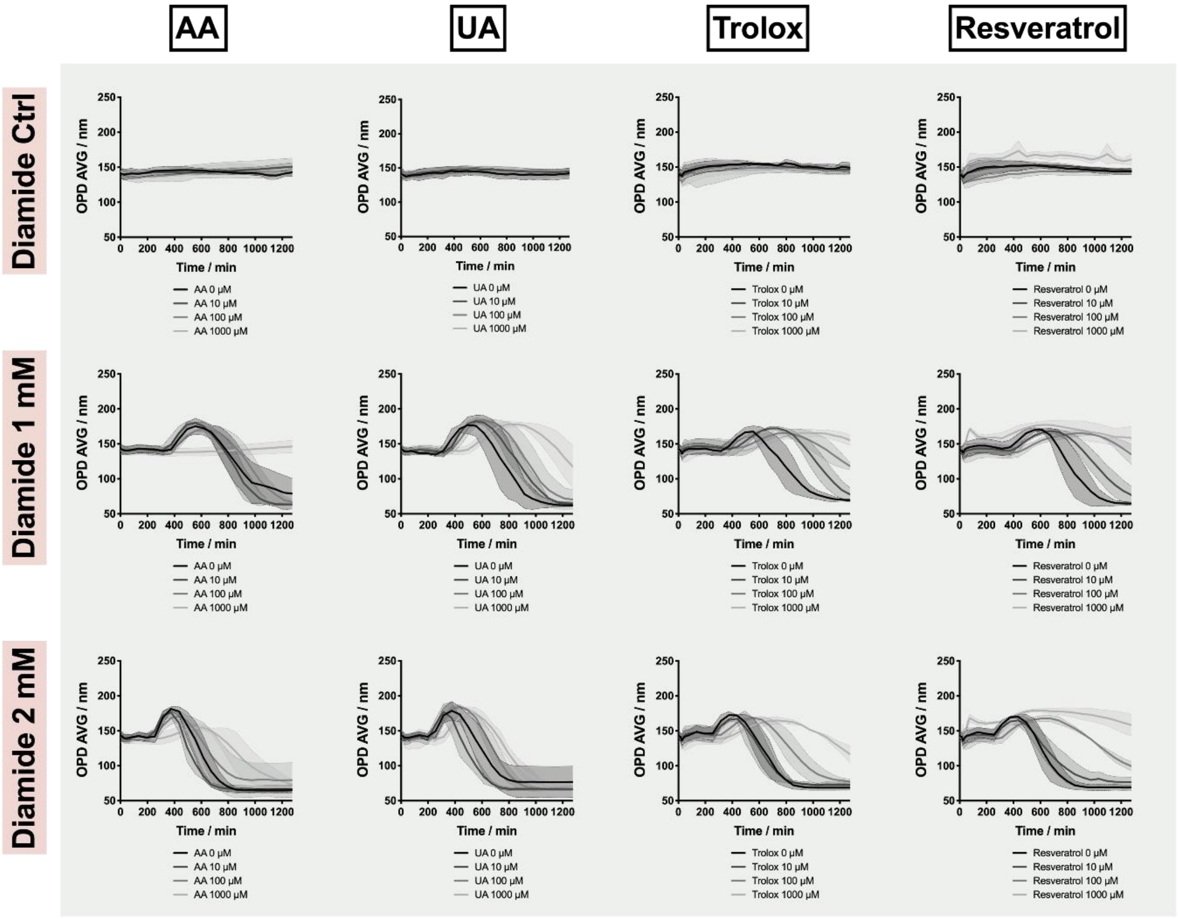

Figure 2. Summary Panel of the Effects on Red Blood Cells (RBCs) of 0, 10, 100 and 1000 μM Ascorbic Acid (AA), Uric Acid (UA), Trolox and Resveratrol against Diamide at Different Concentrations. Timelapse Curves of Average of the Optical Path Difference Distribution (OPD AVG) Parameter Acquired by Digital Holographic Microscopy (DHM). Mean of 3 Red Cell Concentrates with Standard Deviation.

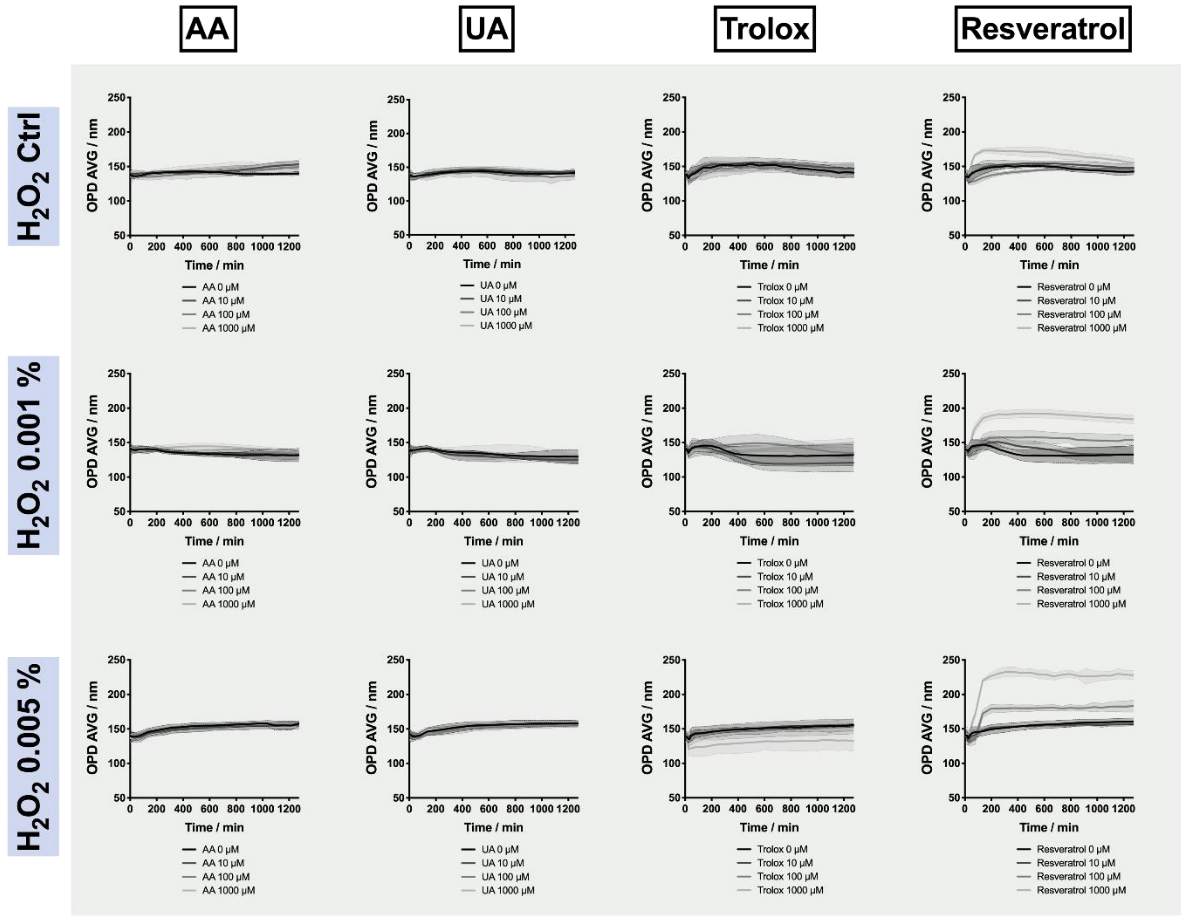

Figure S3. Summary Panel of the Effects on Red Blood Cells (RBCs) of 0, 10, 100 and 1000 μM Ascorbic Acid (AA), Uric Acid (UA), Trolox and Resveratrol against H_2O_2 at Different Concentrations. Timelapse Curves of Average of the Optical Path Difference Distribution (OPD AVG) Parameter Acquired by Digital Holographic Microscopy (DHM). Mean of 3 Red Cell Concentrates with Standard Deviation.

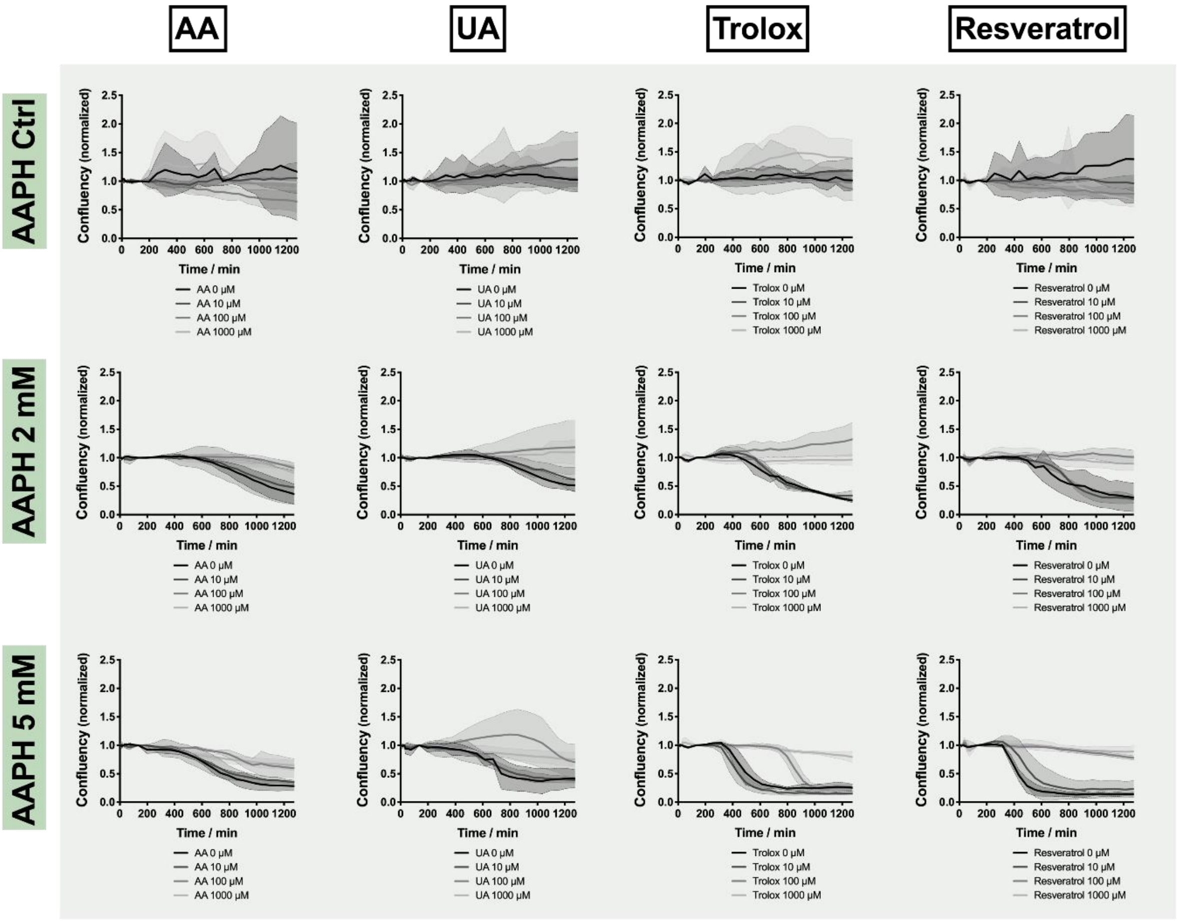

Figure S4. Summary Panel of the Effects on Red Blood Cells (RBCs) of 0, 10, 100 and 1000 μM Ascorbic Acid (AA), Uric Acid (UA), Trolox and Resveratrol against AAPH at Different Concentrations. Timelapse Curves of Normalized Confluency Parameter Acquired by Digital Holographic Microscopy (DHM). Mean of 3 Red Cell Concentrates with Standard Deviation.

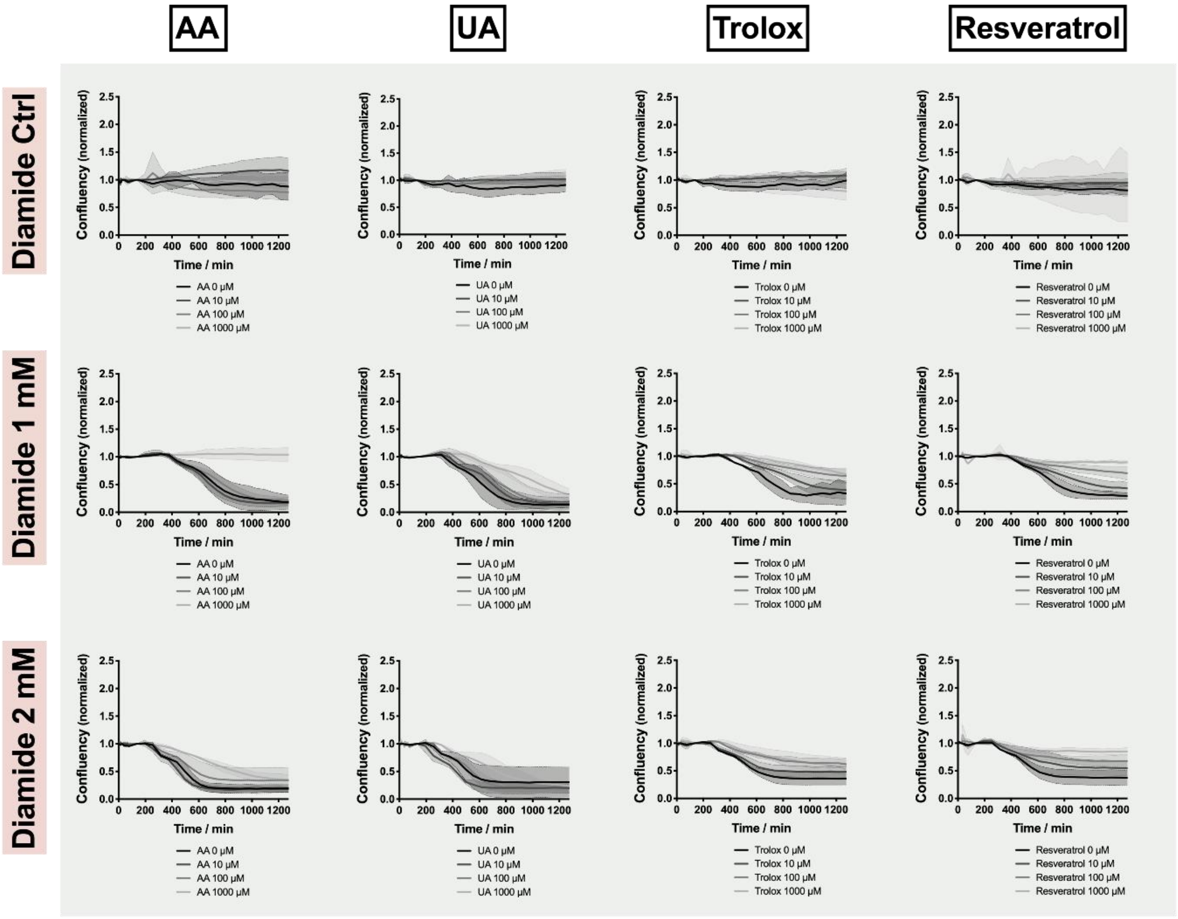

Figure S5. Summary Panel of the Effects on Red Blood Cells (RBCs) of 0, 10, 100 and 1000 µM Ascorbic Acid (AA), Uric Acid (UA), Trolox and Resveratrol against Diamide at Different Concentrations. Timelapse Curves of Normalized Confluency Parameter Acquired by Digital Holographic Microscopy (DHM). Mean of 3 Red Cell Concentrates with Standard Deviation.

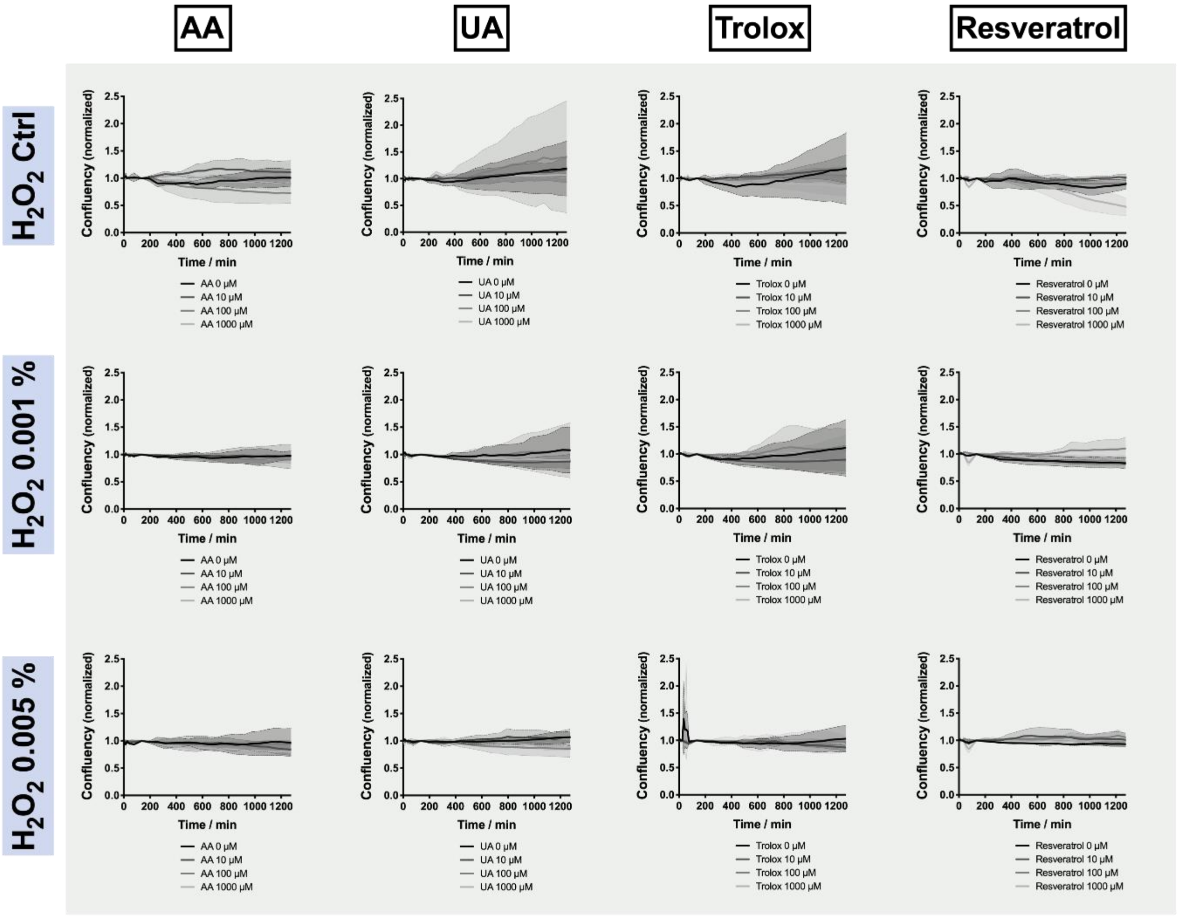

Figure S6. Summary Panel of the Effects on Red Blood Cells (RBCs) of 0, 10, 100 and 1000 µM Ascorbic Acid (AA), Uric Acid (UA), Trolox and Resveratrol against H_2O_2 at Different Concentrations. Timelapse Curves of Normalized Confluency Parameter Acquired by Digital Holographic Microscopy (DHM). Mean of 3 Red Cell Concentrates with Standard Deviation.

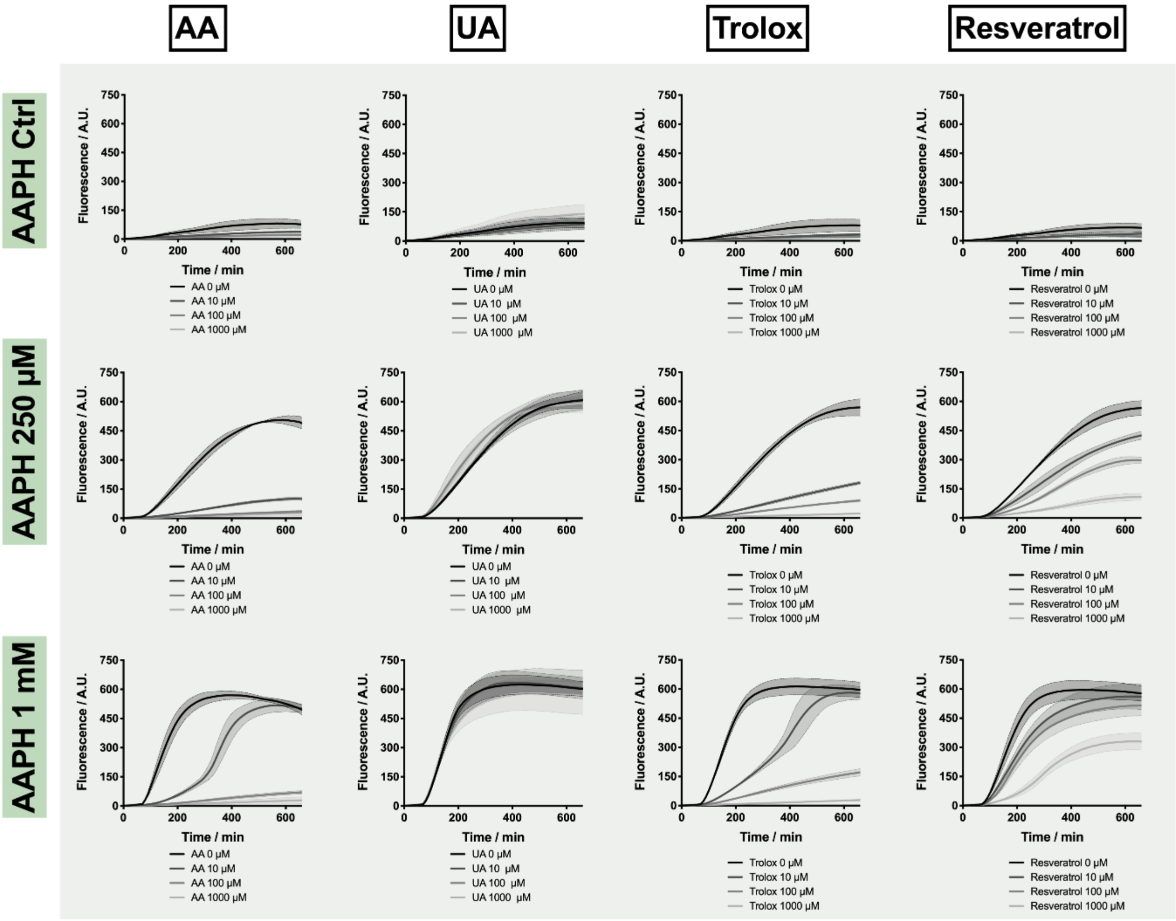

Figure S7. Summary Panel of the Effects on Red Blood Cells (RBCs) of 0, 10, 100 and 1000 µM Ascorbic Acid (AA), Uric Acid (UA), Trolox and Resveratrol against AAPH at Different Concentrations. Timelapse Curves of Fluorescence emission. Mean of 3 Red Cell Concentrates with Standard Deviation.

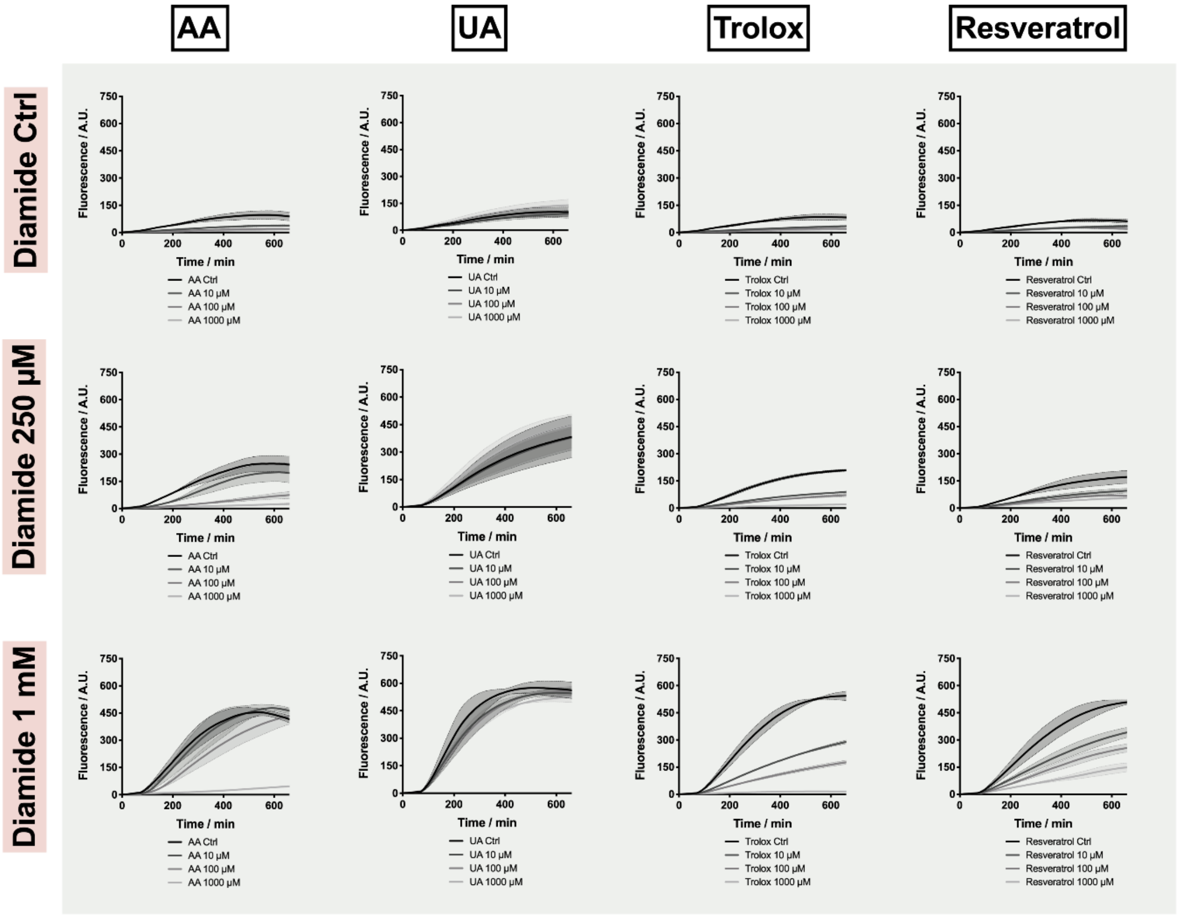

Figure S8. Summary Panel of the Effects on Red Blood Cells (RBCs) of 0, 10, 100 and 1000 μM Ascorbic Acid (AA), Uric Acid (UA), Trolox and Resveratrol against Diamide at Different Concentrations. Timelapse Curves of Fluorescence emission. Mean of 3 Red Cell Concentrates with Standard Deviation.

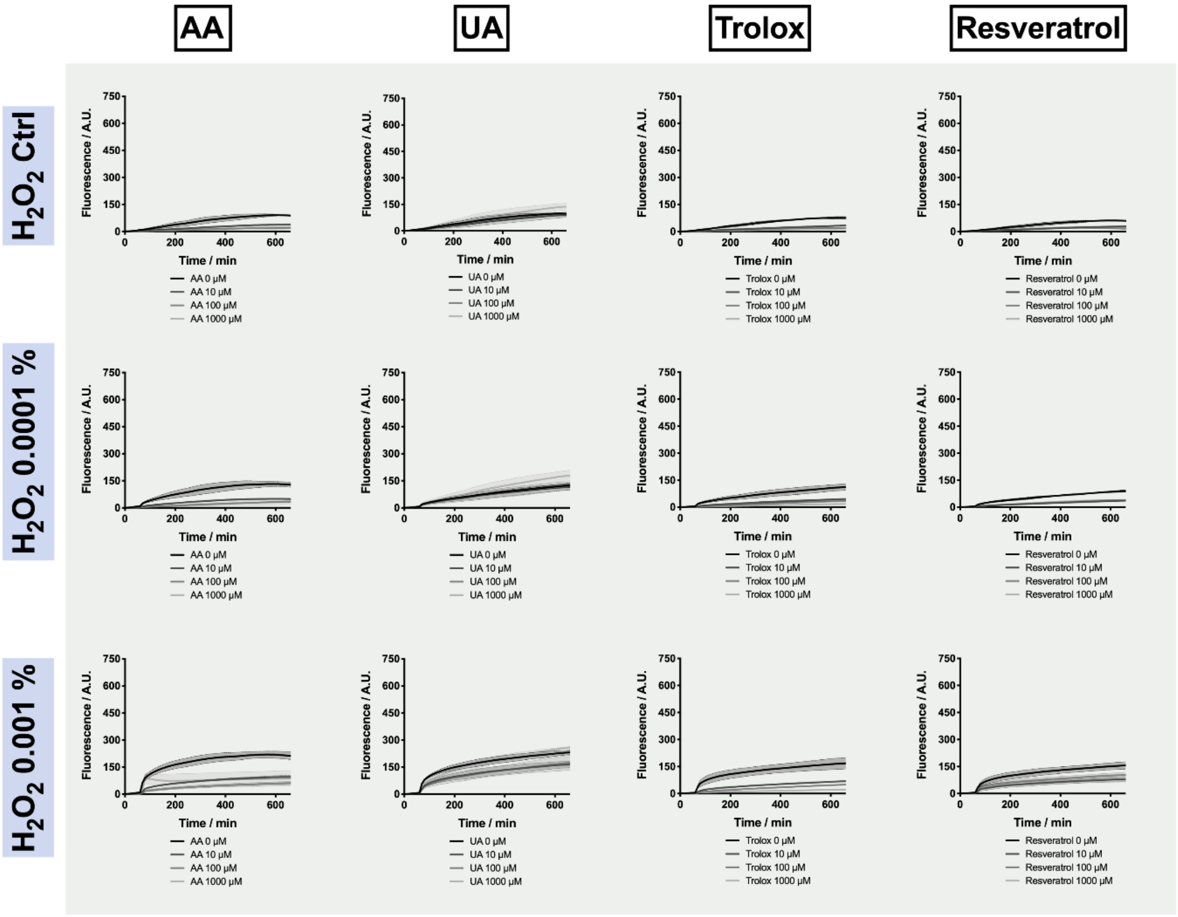

Figure S9. Summary Panel of the Effects on Red Blood Cells (RBCs) of 0, 10, 100 and 1000 µM Ascorbic Acid (AA), Uric Acid (UA), Trolox and Resveratrol against H_2O_2 at Different Concentrations. Timelapse Curves of Fluorescence emission. Mean of 3 Red Cell Concentrates with Standard Deviation.
